# Increased MIB-1 expression in salivary gland pleomorphic adenoma that recurs and undergoes malignant transformation

**DOI:** 10.1038/s41598-022-13082-8

**Published:** 2022-05-30

**Authors:** Anttoni Markkanen, Katri Aro, Anna Ray Laury, Antti A. Mäkitie, Caj Haglund, Timo Atula, Jaana Hagström

**Affiliations:** 1 Department of Pathology, HUSLAB, Helsinki University Hospital and University of Helsinki, PO Box 21, 00014 Helsinki, Finland; 2 Department of Otorhinolaryngology-Head and Neck Surgery, Helsinki Head and Neck Center, Helsinki University Hospital and University of Helsinki, Helsinki, Finland; 3 Department of Mechanical Engineering, Aalto University, Espoo, Finland; 4 Division of Ear, Nose, and Throat Diseases, Department of Clinical Sciences, Intervention, and Technology, Karolinska Institutet and Karolinska University Hospital, Stockholm, Sweden; 5 Research Program in Systems Oncology, Faculty of Medicine, University of Helsinki, Helsinki, Finland; 6 Research Programs Unit, Translational Cancer Medicine, University of Helsinki, Helsinki, Finland; 7 Department of Surgery, University of Helsinki and Helsinki University Hospital, Helsinki, Finland; 8 Department of Oral Pathology and Radiology, University of Turku, Turku, Finland

**Keywords:** Salivary gland diseases, Head and neck cancer, Tumour biomarkers

## Abstract

The objective of this retrospective study was to explore possible changes in histopathological features and expression of cyclin D1 and MIB-1 in salivary gland pleomorphic adenomas (PAs) that recur or undergo malignant transformation. Knowledge of these characteristics might help to guide the management of these rare tumors. The histopathology and immunohistochemical staining characteristics of such tumors were analyzed in a cohort of 65 patients constituting three different groups of tumors: PA, recurrent pleomorphic adenoma (RPA) and carcinoma ex PA (CxPA). The RPAs were divided into two subgroups: primary PA that were known to recur later (PA-prim) and recurrent tumors appearing after a primary tumor (PA-rec). RPAs and CxPAs were compared with PAs without recurrence, which served as a control group. In our study, CxPA and PA-rec, but not PA-prim, showed increased MIB-1 expression compared with the control group. Neither cyclin D1 expression nor any histopathological features showed any association in statistical analyses. CxPA showed increased mitotic activity, squamous metaplasia, and nuclear atypia. Tumor multifocality was more frequent in PA-rec and CxPA. The different MIB-1 expression in CxPA and PA-rec in comparison to PA-prim suggests that the changes in expression could develop after the primary tumor.

## Introduction

Pleomorphic adenoma (PA) is the most common benign salivary gland tumor^1^. If left untreated, PA has the potential to develop into a malignancy in 5–10% of cases^2^. In addition, PA has the potential to recur (recurrent pleomorphic adenoma, RPA) even after adequate surgery, which complicates treatment. The presence of satellite nodules and pseudopodia of the original PA, rupture of the tumor capsule, incomplete removal of the tumor, and close surgical margins may increase the risk of recurrence^3^. Malignant transformation develops more frequently in recurrent tumors^3^. A large Danish nationwide study showed that patients with surgically treated PA developed recurrences in 2.9% of tumors and malignant transformation in 3.3% of recurrent tumors^4^. Malignant PA is usually classified into three subtypes: carcinoma ex pleomorphic adenoma (CxPA), carcinosarcoma (“true malignant mixed tumors”), and metastasizing PA. The most common of these is CxPA, where different histological subtypes can form the malignant component, which makes CxPA a heterogenous group of tumors^5^.

Cyclin D1 is a protein that regulates cells’ progression through the G1-S phase. Its overexpression has been linked to tumorigenesis and malignant progression^6,7^. A recent meta-analysis showed that cyclin D1 overexpression associates with poor prognosis in head and neck cancer patients, and it could thus be a valuable prognostic marker^8^. Ki-67 is a protein that regulates cell proliferation. It is present in multiple phases of the cell cycle, but its expression increases from the late G1 phase to the S phase and is highest at the mitotic phase^9^. Ki-67 is often overexpressed in neoplasms and it represents the proliferation rate of the tumor^10,11^. Its prognostic value as a marker has been studied in many cancers, including head and neck cancer^12,13^. MIB-1 is a monoclonal Ki-67 antibody and it is frequently used in current pathological reporting and to aid in the management of e.g., salivary gland cancer^10^.

Previous studies concerning cyclin D1 and MIB-1 expression in PA, RPA, and CxPA are scarce and show variable results. One study showed increased MIB-1 expression in malignant areas of salivary gland CxPA^14^, while Patel et al. found no difference in cyclin D1 expression in PA and CxPA^15^. Souza et al. reported increased cyclin D1 expression in RPA compared to PA^16^, while another study reported no difference between these tumors^17^.

In this study, we explored the expression of cell cycle regulators cyclin D1 and Ki-67, as well as the histopathological findings of PA, RPA, and CxPA to investigate their role in the recurrent behavior and malignant transformation of these tumors. This knowledge might help us to guide their challenging management. Malignant tumors show changes in their histological appearance and different expression of immunohistochemical markers. Thus, we speculated that these changes could be observed before the malignancy develops. Furthermore, as malignancies develop more commonly in RPA than PA, we hypothesized that there is a histological and immunohistochemical difference between PA and RPA that will help us understand recurrences and malignant transformation.

## Materials and methods

### Ethical approval

The study was approved by the Helsinki University Hospital (HUS) Ethics Committee (Authorization number HUS/967/2017), and an institutional study permission was granted (§41/2017). All methods were performed in accordance with the relevant guidelines and regulations.

### Tissue samples and histopathology

All tumor samples were re-evaluated for the following histopathological features: cell/stroma ratio, stromal composition (myxoid, chondroid, hyalinized, fat tissue), percentage of ductal structures, squamous metaplasia, mucous cells, sebaceous differentiation, oncocytic differentiation, mitotic features, and nuclear atypia. In addition, we analyzed the presence of tumor capsule, margin positivity, tumor diameter, tumor infiltration (budding) into the capsule, and multifocality. Single-author-analyses were first carried out (A.M.), and then experienced head and neck pathologists (A.L. and J.H.) re-evaluated the results.

### Immunostainings

We collected all available tumor blocks from the study groups, based on their diagnostic slides. To prepare the tumor samples for immunohistochemical staining, we used the following protocol for both cyclin D1 and MIB-1: 4 µm thick slides were prepared from formalin-fixed paraffin-embedded (FFPE) tumor blocks, deparaffinized in xylene, and rehydrated with ethanol. Antigen retrieval was carried out by heat induced epitope retrieval (HIER), and the retrieval solution was pH 9, 15 min 98 °C. After that we deployed blocking of endogenous peroxidase and added primary antibody. For cyclin D1 we used the monoclonal rabbit anti-cyclin D1 antibody (SP4), ab 16663, Abcam with incubation time of O/N +5. For MIB-1, Dako Agilent monoclonal mouse antibody Ki-67/MIB-1 7240 (Dako Agilent, Santa Clara, USA) was used with incubation time of O/N +5. Secondary antibody (EnVision Flex, Dako, Clostrup, Denmark), chromogen (EnVision Flex DAB, Dako, Clostrup, Denmark) and substrate (Dako Mayer’s Hematoxylin, Dako, Clostrup, Denmark) were deployed. The staining process was performed with an Autostainer 480S (LabVision, UK).

### Scoring

Immunopositivity in the tumor samples was scored by two investigators (A.M. and J.H.) who had no knowledge of the clinicopathological data. Nuclear and cytoplasmic expression of cyclin D1 were scored separately for each sample. Scoring of cyclin D1 expression was based on the percentage of nuclear and cytoplasmic immunopositivity in tumor cells. Scoring was as follows: negative (0), 0–10% expression; weak positivity (1), 11–40% expression; moderate positivity (2), 41–70% expression; and strong positivity (3), 71–100% expression. We used the following scale for MIB-1 expression: negative (0), 0–4% expression; weak positivity (1), 5–10% expression; moderate positivity (2), 11–20% expression; and strong positivity (3), > 20% expression. Breast tissue was used as a positive control for both cyclin D1 and MIB-1.

### Patient selection and source of data

The electronic pathology archives and hospital patient records of the Helsinki University Hospital served as the source of data. We constituted three different main groups for comparison: “conventional” pleomorphic adenomas (PA) which served as the control group, recurrent pleomorphic adenomas (RPA), and carcinoma ex pleomorphic adenomas (CxPA). In the RPA and CxPA groups, we included patients who had a tumor in any major or minor salivary gland during the period 2000–2018.

The first group served as a control group and consisted of parotid gland PAs that had been treated adequately and showed no signs of recurrence within a 12-year follow-up. To achieve a long (minimum of 12 years) follow-up and assurance that the tumor had not recurred, we selected consecutive patients treated through 2005–2006. We included patients with parotid gland tumors who underwent adequate treatment, either superficial or partial parotidectomy, with no report of capsule rupture and who presented with a tumor ≥ 1.0 cm in diameter, to achieve enough material for histopathological analysis. We sent a questionnaire in a preaddressed, prepaid envelope to all patients to confirm their status at the time when data were retrieved, and to verify possible later contacts with any healthcare unit due to a salivary gland tumor. All 27 patients who fulfilled these criteria responded, and none reported health care visits or sequalae regarding the operated site.

In the RPA group, we included only recurrent tumors that appeared after an adequately treated primary tumor, i.e., those after superficial parotidectomy (for parotid gland tumors) and with no capsular rupture. Also, in this group we included only patients without evidence of a salivary gland malignancy during follow-up. We presumed that these selection criteria would best represent and identify tumors that are intrinsically prone to recur. Altogether 20 patients fulfilled these criteria and they experienced 27 recurrent events in total. Of these, six patients had a second recurrence, and one patient developed a third recurrence.

For histological and immunohistochemical studies, the tumors within the RPA group were split into two subgroups according to the sample investigated: primary PAs that were later known to recur (PA-prim) and recurrent tumors that appeared after the primary tumor (PA-rec). These subgroups were formed to study the clinical nature of the primary tumors in the PA-prim group compared to the control group more specifically, and to reveal potential histopathological changes in the tumor before it recurred.

**Figure 1:**
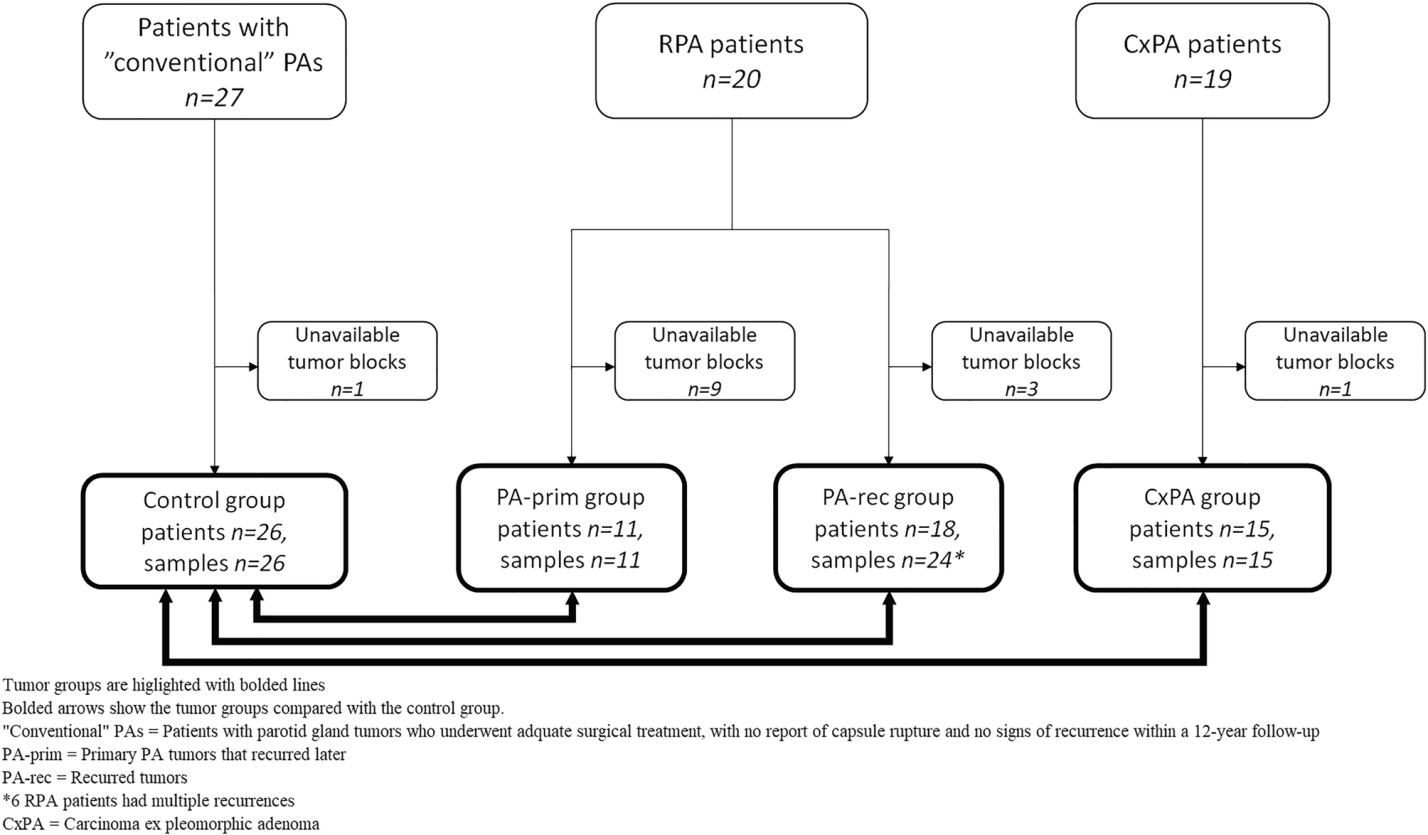
Flowchart illustrating the formation of the tumor groups.

In total, the PA-prim and the PA-rec groups contained tumors from 19 of the 20 RPA patients, because both the primary and the recurrent tumor blocks from one RPA patient were unavailable (Fig. 1). Tumor blocks were available from 11 PA-prim tumors from 20 patients. The cases with unavailable tissue samples had been treated elsewhere, and some of them decades ago. In the PA-rec group, 24 tumor blocks were available from 18 patients, some of whom had multiple recurrences. Tumor blocks were available from 15 out of the 19 CxPA cases, and from 26 out of the 27 cases in the control group. Table 1 further clarifies the division of RPA subgroups.

**Table 1:**
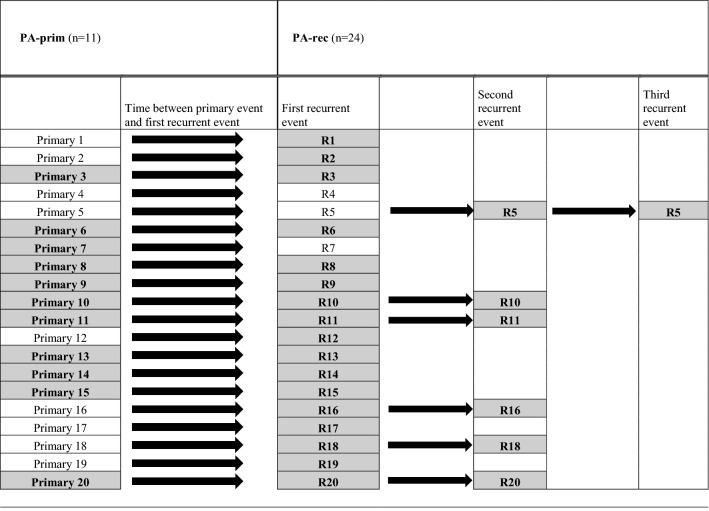
Formation of the subgroups of RPA patients, n = 20.

The number represents individual patients, n = 20.

Available tumor blocks are highlighted in gray/bolded, and these formed the final study population.

PA-prim = Primary tumors of RPA patients.

PA-rec = Recurrent tumors of RPA patients.

The CxPA group included 19 patients. Twelve of these patients were diagnosed de novo, whereas in seven patients, the malignancy occurred in RPA (one in the first recurrence, six in the second or later recurrence). None of the patients were included in both the CxPA and RPA groups.

### Statistical analysis

The Mann–Whitney U test was used to compare scoring for expression of cyclin D1 and MIB-1 between the control group, RPA groups, and CxPA group as a pair comparison. The same test was used to compare the histological parameters with ordinal values. The Pearson chi-squared test was used to compare nominal values in suitable clinical and histological features. *p* value < 0.05 was considered statistically significant. Statistical analyses were performed using SPSS® software (version 27, IBM®).

## Results

### Clinical and histopathological features

Clinical features of the entire cohort are shown in Table 2. Patients were more frequently female in both the control (19 out of 27; 70%) and RPA (15 out of 20; 75%) groups, but males predominated in the CxPA group (12 out of 19; 63%). The median age at diagnosis was 48 in the control group, and 60 in the CxPA group. Median time between the primary tumor and the first recurrence in the RPA group was 10.5 years, with a wide range of 1–25 years.

**Table 2:** Clinical information of patients' groups.

Group	Control (n = 27)	RPA (n = 20)**	CxPA (n = 19)
**Gender**			
Male	8	5	12
Female	19	15	7
Age (years)*			
Median	48	40	60
Range	10–68	17–66	26–76
**Tumor site**			
Parotid gland	27	16	13
Submandibular gland	0	3	5
Minor salivary glands	0	0	1
Parapharyngeal space	0	1	0
**Time between the primary tumor and the first recurrence (years)**			
Median	–	10.5	–
Range	–	1–25	–

RPA = Patients with recurrent pleomorphic adenoma.

CxPA = Patients with carcinoma ex pleomorphic adenoma.

*Patients' age at time of diagnosis.

**Information available from 18/20 patients.

Tumor multifocality was more frequent in the PA-rec group (n = 10; 42%; *p* = 0.015) and CxPA (n = 4; 27%; *p* = 0.031) compared with the control group (n = 1; 3.8%). The PA-prim group included no multifocal tumors (Table 3).

**Table 3:** Tumor-related information in the investigated groups.

Parameters	Control (n = 26)	RPA			CxPA (n = 15) **	
		PA-prim (n = 11)	PA-rec (n = 24)			
**Tumor capsule**						
Present	26	11	23			
Absent	0	0	1			
**Margin**						
Positive	0	0	1		2	
Negative	26	11	23		13	
**Tumor diameter (mm)**						
Mean	38	44	28		52	
Range	13–65	30–55	8–75		18–110	
**Infiltration to capsule (budding)**						
Present	19	9	22		–	
Absent	7	2	2		–	
**Multifocality**						
Multifocal	1	0	10		4	
Single tumor	25	11	14	0.015*	11	0.031*

*Pearson- chi square, Asymp. Sig. (2-sided). Bolded value is statistically significant (*p* < 0.05).

** Ruptured capsule and/or tumor budding were not present or measured due to the tumor invasion.

RPA = Patients with recurrent pleomorphic adenoma.

CxPA = Patients with carcinoma ex pleomorphic adenoma.

PA-prim = Primary tumors of RPA patients.

PA-rec = Recurrent tumors of RPA patients.

**Figure 2:**
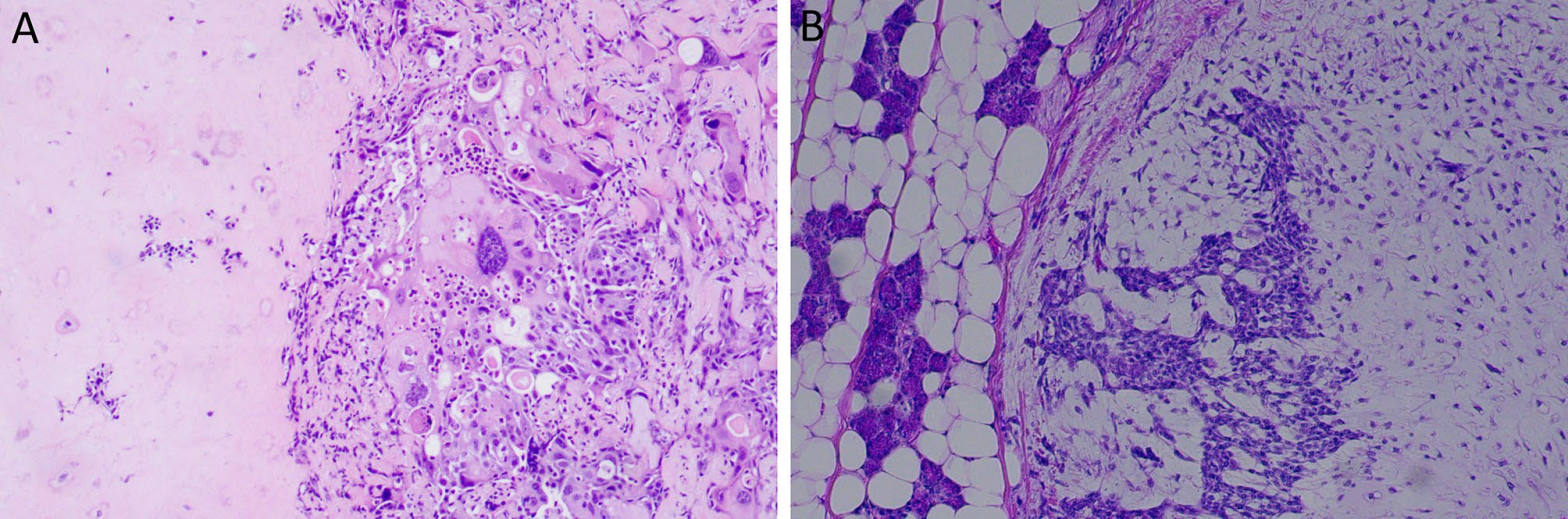
(**A**) Nuclear atypia and chondroid tissue formation in CxPA Magnification ×200. (**B**) Myxoid stroma and capsuled PA surrounded by healthy parotid tissue. Magnification ×100.

CxPAs had more mitotic figures compared with the control group (*p* < 0.001; Table 4). Both the PA-prim and PA-rec groups had low mitotic activity, like the control group. In addition, CxPAs presented with higher nuclear atypia compared with the control group (*p* = 0.001). Higher nuclear atypia was present in the PA-rec tumors more often than in the control group, but this difference did not reach significance (*p* = 0.066) since only three out of 24 PA-rec tumors showed moderate nuclear atypia and none of the tumors showed high atypia. All tumors in the PA-prim and control groups had only mild nuclear atypia. Squamous metaplasia was more frequently present in CxPA compared with the control group (*p* = 0.036; Table 4; Fig. 2).

**Table 4:** Histological parameters and comparison with the control group.

Parameters	Control (n = 26)	RPA				CxPA (n = 15)	*p* value
		PA-prim (n = 11)	*p* value	PA-rec (n = 24)	*p* value		
**Cell/stroma ratio (%)**							
< 20	4	4		1		0	
20–59	13	2		11		4	
60–80	5	3		5		5	
> 80	4	2		7		6	
**Ductal structures (%)**							
0–19	18	10		18		11	
20–39	4	1		4		4	
40–59	3	0		1		0	
60–79	0	0		0		0	
≥ 80	1	0		1		0	
**Stroma**							
Myxoid	24	9		20		9	
Chondroid component	1	2		3		1	
Hyalinized	0	0		0		4	
Myoepithelial	0	0		0		0	
Absent or minor	1	0		1		1	
Fat tissue	0	0		0		0	
Mitotic activity*							
0–2	25	11		23		7	
3–5	1	0		1		4	
6–8	0	0		0		3	
> 8	0	0		0		1	
							< 0.001**
**Nuclear atypia**							
Mild	25	11		21		5	
Moderate	0	0		3		7	
High	0	0		0		3	
					0.066**		< 0.001**
**Oncocytic differentiation**							
Present	1	0		2		1	
Absent	25	11		22		14	
**Sebaceous differentiation**							
Present	0	0		0		0	
Absent	26	11		24		15	
**Mucous cells**							
Present	0	0		1		0	
Absent	26	11		23		15	
**Squamous metaplasia**							
Present	1	0		2		5	
Absent	25	11		22		10	
							0.036***

*Consecutive 10 HPF (high power fields).

**Mann–Whitney U, bolded *p* values are statistically significant (*p* < 0.05).

***Pearson Chi-Squared, bolded *p* values are statistically significant (*p* < 0.05).

RPA = Patients with recurrent pleomorphic adenoma.

CxPA = Patients with carcinoma ex pleomorphic adenoma.

PA-prim = Primary tumors of RPA patients.

PA-rec = Recurrent tumors of RPA patients.

### MIB-1

**Figure 3:**
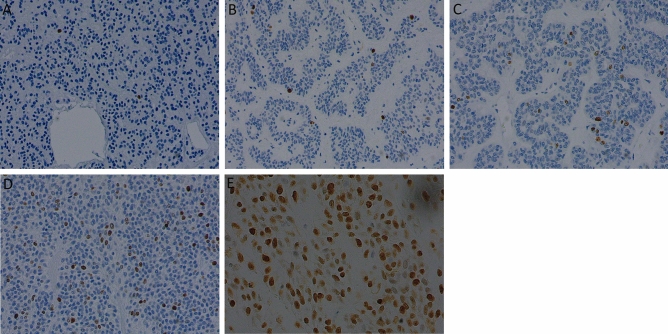
(**A–C**) Increasing MIB-1 proliferation index in consecutive PA recurrencies. (**A**) primary PA; (**B**) first recurrence; (**C**) later recurrence. (**D**) High MIB-1 in CxPA. (**A**–**D**) magnification ×200. (**E**) Nuclear cyclin D1 expression in CxPA. Magnification ×400.

CxPA showed significantly higher MIB-1 positivity compared with the control group (*p* < 0.001), as eight out of 15 showed ≥ 20% MIB-1 expression and only two tumors had an expression < 5% (Table 5, Fig. 3D). Additionally, the PA-rec group had higher MIB-1 expression compared with the control group (*p* = 0.031). The PA-prim group and the control group showed mostly low (< 5%) MIB-1 expression (Table 5). One RPA patient with multiple recurrent events showed increasing MIB-1 expression in every subsequent recurrent tumor (Fig. 3A–C). This tendency, however, was not observed in other patients who experienced multiple recurrences.

**Table 5:** Expression of cyclin D1 and MIB-1 in the investigated groups and the controls.

Variables	Control (n = 26)	PA-prim (n = 11)	*p* value	PA-rec (n = 24)	*p* value	CxPA (n = 15)	*p* value
**CyclinD1, nuclear**							
Negative	2	2		2		0	
Weakly positive	11	3		7		6	
Moderately positive	9	5		10		6	
Strongly positive	4	1		5		3	
			0.736*		0.583*		0.478*
**CyclinD1, cytoplasmic**							
Negative	12	8		12		6	
Weakly positive	12	3		9		6	
Moderately positive	2	0		3		3	
Strongly positive	0	0		0		0	
			0.122*		0.949*		0.529*
**MIB-1 (%)**							
0–4	25	11		18		2	
5–10	1	0		4		5	
11–20	0	0		2		4	
> 20	0	0		0		4	
			0.515*		0.031*		< 0.001*

*Mann–Whitney U, Asymp. Sig (2-tailed).

Bolded *p* values are statistically significant. Statistically significant *p* value *p* < 0.05.

RPA = Patients with recurrent pleomorphic adenoma.

CxPA = Patients with carcinoma ex pleomorphic adenoma.

PA-prim = Primary tumors of RPA patients.

PA-rec = Recurrent tumors of RPA patients.

### Cyclin D1

All tumor groups showed both nuclear and cytoplasmic expression of cyclin D1, and the expression varied between tumors within the same group (Table 5). On average, nuclear expression of cyclin D1 was stronger than cytoplasmic in all groups. Both nuclear and cytoplasmic expression of cyclin D1 appeared to be elevated in the PA-rec and CxPA groups compared with the control group, but the difference remained statistically insignificant. In addition, cytoplasmic cyclin D1 appeared to be lower in the PA-prim group than in the control group, but without statistical significance (*p* = 0.122). While the variation of cyclin D1 expression was high between tumors within every tumor group, six out of 15 CxPAs showed moderate or high nuclear cyclin D1 expression. All these six CxPAs had developed after at least one recurrent event (Table 5, Fig. 3E). Three of the 12 CxPA tumors originating from primary PA tumors showed moderate or high nuclear cyclin D1 expression.

Neither cyclin D1 nor MIB-1 expression showed any correlation with the clinical parameters or with each other.

## Discussion

In the present study, both PA-rec and CxPA showed increased MIB-1 expression. Interestingly, primary tumors of RPA patients did not express this feature, which might suggest that MIB-1 expression increases in recurrent events and in malignant transformation. This leads us to speculate whether the recurrent behavior and malignant transformation in PA are linked, and if a recurrent event increases the probability of a PA to develop into a malignancy, even though CxPA can originate from a nonrecurrent PA. Previous studies have also shown a similar observation that multiple recurrencies can lead to malignant transformation^18^. Additionally it is widely known that MIB-1 overexpression is linked to malignant tumors^19^.This is in line with our results since recurrent PA and CxPA showed higher proliferation index when compared with primary PA. However, MIB-1 expression does not seem to be a suitable marker for predicting a later recurrence or malignant behavior since it was not increased in the primary tumors of RPA patients. In our series cyclin D1 expression showed no differences between the groups although CxPAs that were developed after a preceding RPA showed higher nuclear expression of cyclin D1. In addition, histopathological features showed no differences between PA-prim group and the “conventional” PAs, which further suggests that PA-prims differ from their later recurrent tumors.

In our series, the histopathological features of PA, RPA and CxPA showed wide heterogeneity, as Singh et. al. also reported in their study on CxPA^20^. In our study, CxPA exhibited increased mitotic activity and higher levels of nuclear atypia compared with PA or RPA. Our finding is in line with a review by Antony et al., which describes similar findings in CxPA^21^. Additionally, in our cohort, CxPAs exhibited a higher amount of squamous metaplasia. Still, the tumors within these groups presented individual variation in the percentage of ductal structures, cell/stroma ratio, and stromal composition. The individual variation of histological features hinders their utilization in predicting recurrent or malignant behavior. However, the PA-rec group showed some exceptions: multifocal disease was more frequent in PA-rec and CxPA, as was also shown in a previous study by Witt et al.^18^. Additionally, in our series PA-rec tumors showed moderate nuclear atypia only in three cases, of which two were from the same patient. These observations, especially tumor multifocality, suggest that CxPA and PA-rec tumors might share some similarities in their histopathological appearance.

In our study, MIB-1 expression was higher in CxPA and in PA-rec compared with PA. Previously, Larsen et al. reported increased MIB-1 expression in salivary gland malignancies^22^. In our series, one patient had three subsequent recurrent events and those tumors showed a constant increase in MIB-1 expression in every subsequent recurrence. Thus, it is tempting to hypothesize that elevated expression of this proliferation marker could indicate whether RPA is more prone to recur and develop a malignancy, although Glas et al. reported no such tendency^17^. The PA-prim group in our cohort did not express increased levels of MIB-1, which suggests that increased MIB-1 expression seems to appear after recurrence and could be part of the development of the recurrence. Our control group and PA-prim group showed similar histological and immunohistochemical characteristics, which further supports this. Still, the intrinsic or extrinsic factors that trigger the primary tumors to recur later remain unknown.

Cyclin D1 expression showed no statistically significant difference between PA-prim, PA-rec, or CxPA compared to the control group. Our findings are in line with those reported by Patel et al., who compared the expression of cyclin D1 in 29 cases of PAs and 14 CxPAs^15^, but their study did not include RPA tumors. Another study by Souza et al. showed increased cyclin D1 expression in RPA tumors compared with PA in a series of 24 PAs and 21 RPAs. Their series contained also two CxPAs, which showed cyclin D1 positivity^16^. In comparison to previous studies, we split the RPA tumors into two subgroups, which can explain some of the differences with other reports. Interestingly, our study showed that CxPA that developed after a preceding recurrent tumor (six tumors out of 15) showed moderate or high nuclear cyclin D1 expression compared with other CxPAs. It is speculated whether this characteristic might lead to an enhancement in neoplastic growth^23^. On the other hand, another study containing multiple different benign and malignant salivary gland tumors showed no correlation between cyclin D1 expression and biological behavior of the tumor^24^. While overexpression of cyclin D1 has been linked to malignant tumors^25^, this seems not to be the case with salivary gland tumors. We hypothesize that this is particularly due to the heterogenous nature of salivary gland tumors. Therefore, the role of cyclin D1 in recurrent and malignant behavior of PA remains unclear. Ramos-García et. al reported increased cytoplasmic cyclin D1 expression to be associated with advanced tumor stage and presence of invasive cell morphology in squamous cell carcinomas, and in addition an association between nuclear and cytoplasmic expression^26^. Elevated nuclear cyclin D1 expression might play a role in malignant transformation but this requires further studies in larger series that include information from the preceding RPA tumors.

The limitations of our study include the rarity of salivary gland tumors in general, which hinders the availability of a large sample size. While PA is the most common tumor of the salivary gland, its malignant form is uncommon. Furthermore, information regarding PA-prim was limited, as many of them had been treated several years or even decades ago or elsewhere, and thus their tumor blocks were unavailable for this study. Strengths of our study include its population-based setting and long follow-up time. We were able to accurately form specific selection criteria with the aim of gathering a series that would adequately represent the biological behavior of each tumor group. Even though we set the inclusion criteria for each group, our patient series was drawn from unselected populations. The recurrent tumors, CxPAs and the control PAs were treated at only one institute.

Adequate surgery at the primary stage is the most important issue to avoid tumor recurrences. Surgery of salivary gland tumors requires special expertise, and therefore we have nowadays centralized the management of these tumors to one unit and to only a few surgeons within our catchment area. In our institute we do not follow up benign PAs after surgery. Nevertheless, despite adequate surgery, some PAs do recur. Treatment of RPA is even more challenging, as it may also require sacrifice of the facial nerve^3^. Among elderly patients or those with many comorbidities, salivary gland tumors are sometimes left untreated if they are regarded as benign PAs e.g., by cytological means. To choose proper management and to inform patients better, there is a need to find out what predicts the behavior of these tumors and to discover specific markers that can predict the likelihood of possible malignant transformation.

## Conclusion

Our study showed increased MIB-1 expression in CxPA and RPA, which might suggest that these tumors share similarities in recurrent potential and malignant transformation. However, increased MIB-1 expression was not found in the primary tumors of RPA patients, indicating that changes appear later during tumor progression. Thus, MIB-1 does not seem to be usable in predicting recurrent behavior of PA at the primary stage. PA-prims and “conventional” PAs showed no differences regarding histopathological features. Cyclin D1 showed no difference in the expression in any group of tumors investigated in our study.

## Data Availability

The datasets generated during and/or analyzed during the current study are not publicly available due to the sample size and rarity of these tumors. Data are available from corresponding author on reasonable request.
