# Author Correction: Increased MIB-1 expression in salivary gland pleomorphic adenoma that recurs and undergoes malignant transformation

**DOI:** 10.1038/s41598-022-15267-7

**Published:** 2022-06-27

**Authors:** Anttoni Markkanen, Katri Aro, Anna Ray Laury, Antti A. Mäkitie, Caj Haglund, Timo Atula, Jaana Hagström

**Affiliations:** 1 Department of Pathology, HUSLAB, Helsinki University Hospital and University of Helsinki, PO Box 21, 00014 Helsinki, Finland; 2 Department of Otorhinolaryngology-Head and Neck Surgery, Helsinki Head and Neck Center, Helsinki University Hospital and University of Helsinki, Helsinki, Finland; 3 Department of Mechanical Engineering, Aalto University, Espoo, Finland; 4 Division of Ear, Nose, and Throat Diseases, Department of Clinical Sciences, Intervention, and Technology, Karolinska Institutet and Karolinska University Hospital, Stockholm, Sweden; 5 Research Program in Systems Oncology, Faculty of Medicine, University of Helsinki, Helsinki, Finland; 6 Research Programs Unit, Translational Cancer Medicine, University of Helsinki, Helsinki, Finland; 7 Department of Surgery, University of Helsinki and Helsinki University Hospital, Helsinki, Finland; 8 Department of Oral Pathology and Radiology, University of Turku, Turku, Finland

Correction to: Scientific Reports https://doi.org/10.1038/s41598-022-13082-8, published online 30 May 2022

The original version of this Article omitted an affiliation for Jaana Hagström. The correct affiliations are listed below.

Department of Pathology, HUSLAB, Helsinki University Hospital and University of Helsinki, PO Box 21, 00014, Helsinki, Finland

Research Programs Unit, Translational Cancer Medicine, University of Helsinki, Helsinki, Finland

Department of Oral Pathology and Radiology, University of Turku, Turku, Finland

The original Article has been corrected.

